# Identification of the Chemical Constituents in Simiao Wan and Rat Plasma after Oral Administration by GC-MS and LC-MS

**DOI:** 10.1155/2017/6781593

**Published:** 2017-05-25

**Authors:** Yunshuang Fan, Yamei Li, Yuanyuan Wu, Lixin Li, Yuming Wang, Yubo Li

**Affiliations:** ^1^State Key Laboratory of Separation Membranes and Membrane Processes, Tianjin Polytechnic University, Tianjin 300387, China; ^2^School of Environmental and Chemical Engineering, Tianjin Polytechnic University, Tianjin 300387, China; ^3^School of Traditional Chinese Materia Medica, Tianjin University of TCM, No. 312, Anshan West Road, Nankai District, Tianjin 300193, China; ^4^Tianjin State Key Laboratory of Modern Chinese Medicine, No. 88, Yuquan Road, Nankai District, Tianjin 300193, China; ^5^Tianjin International Joint Academy of Biomedicine, No. 220, Dongting Road, Tianjin Economic-Technological Development Area, Tianjin 3000457, China

## Abstract

Simiao Wan (SMW), an important multiherbal formula used in traditional Chinese medicine, is extensively used to treat rheumatoid arthritis. However, the knowledge of the bioactive components of SMW remains unclear. Thus, gas chromatography–mass spectrometry (GC-MS) and liquid chromatography–mass spectrometry (LC-MS) were used to analyze the chemical constituents of volatile and nonvolatile extracts of SMW, as well as its absorbed components in rat plasma after oral SMW administration. Identification of several compounds was enabled by comparison of retention times, MS spectra, and MS/MS spectral data with the standard substance and reference materials reported in the literature. In the volatile extracts, GC-MS identified 26 compounds in vitro, three of which observed in blood by GC-MS. In the nonvolatile extracts, LC-MS identified 49 compounds in SMW; 18 compounds containing 7 prototype compounds, 5 metabolites, and 6 unknown compounds were absorbed by blood. The proposed GC-MS and LC-MS method was appropriate not only for the rapid screening and identification of multiple components of an SMW extract but also for screening its bioactive constituents in vivo. The proposed method could be a promising tool for the quality control of other Chinese herbal medicines.

## 1. Introduction

Simiao Wan (SMW), first described by Zhang Bingcheng in the Qing Dynasty, is an important multiherbal formula used in traditional Chinese medicine (TCM). The formula consists of four herbs, namely,* Cortex Phellodendri Chinensis, Rhizoma Atractylodis, Achyranthes bidentata*, and* Semen Coicis*, and has been extensively used to treat rheumatoid arthritis (RA). Pharmacological studies have shown that SMW decreases the expression of several inflammatory factors, including IL-1*β*, IL-6, and TNF-*α* mRNA [1], SMW has attracted considerable attention worldwide because of its high effectiveness against RA and low toxicity [2].

The chemical constituents of SMW extracts are rather complex, and determination of the functional role of each component in vivo requires identification of the active constituents of the formula. Traditional chemical separation methods for screening bioactive components are laborious, time-consuming, and often provide unreliable results. Thus, determination of a simple, rapid, and reliable method with which to carry out high-throughput screening of active constituents in Chinese herbal formulas is necessary. In recent years, developments in the field of plasma pharmacochemistry [3] have suggested that the effective constituents of Chinese herbal formula can be determined by analyzing compounds absorbed by blood after oral administration [4]. Plasma pharmacochemistry, a field of study that offers a new method for screening active components from TCM, may provide a link between chemical components and clinical effects.

Gas chromatography–mass spectrometry (GC-MS) and liquid chromatography–mass spectrometry (LC-MS) have been successfully applied in the study of chemical constituents in vitro and plasma pharmacochemistry in vivo [5]. In the present study, both GC-MS and LC-MS are used to analyze the chemical constituents of SMW and their metabolites in rat plasma. The study establishes a relatively comprehensive and systematic method to the analysis of SMW. The results provide important groundwork for future clinical studies and are valuable for the quality control of Chinese medicinal formulas.

## 2. Materials and Methods

### 2.1. Reagents and Materials

Acetonitrile and methanol (HPLC grade) were obtained from Merck Co. (Darmstadt, Germany), while formic acid (HPLC grade) was purchased from Sigma (St. Louis, MO, USA). The deionized water used in the experiments was purified by a Milli-Q system (Millipore, Bedford, MA, USA). All other reagents and chemicals used were of analytical grade.

Berberine hydrochloride, palmatine hydrochloride, and limonin reference standards were purchased from the National Institute for the Control of Pharmaceutical and Biological Products (Beijing, China). Magnoflorine was purchased from Tianjin Zhongxin Pharmaceuticals Company (Tianjin, China). Obacunone was purchased from the Analysis and Test Center in Shandong Province (Shandong, China), ecdysterone was purchased from Shenzhen Mei Ho Biotechnology Co., Ltd. (Shenzhen, China), *β*-eudesmol, and atrctylodin were provided by the Shanghai Research Center of Chinese Standardization (Shanghai, China). All reference compounds had above 98% purity as determined by HPLC analysis.

*Phellodendri Amurensis Cortex, Atractylodis Lanceae Rhizoma, Achyranthis Bidentatae Radix*, and* Semen Coicis* were purchased from herbal markets in China and authenticated at the Department of Experimental Teaching, Tianjin University of Traditional Chinese Medicine (Tianjin, China).

### 2.2. Preparation of Plant Extracts

#### 2.2.1. Volatile Extracts

According to the weight ratio described in* Chinese Pharmacopoeia* (2005 edition), 60 g SMW was accurately weighed and extracted with 1200 mL water for 6 h by steam distillation. The volatile extracts were removed from water with anhydrous sodium sulfate, after which 0.2 mL of the volatile extracts was dissolved in 2 mL ethyl acetate and filtered through a 0.22 *μ*m membrane before GC-MS analysis. Similarly, 20 g* Cortex Phellodendri*, 10 g* Rhizoma Atractylodis*, 10 g* Radix Achyranthis Bidentatae*, and 20 g* Semen Coicis* were processed to acquire volatile extracts as above.

#### 2.2.2. Nonvolatile Extracts

SMW (7.5 g),* Cortex Phellodendri* (2.5 g),* Rhizoma Atractylodis* (1.25 g),* Radix Achyranthis Bidentatae* (1.25 g), and* Semen Coicis* (2.5 g) were ground and steam distilled in 150 mL water for 6 h. The water extracts were filtered, and the residues were ultrasonically extracted using 50 mL of 75% ethanol at room temperature for 1 h. Two batches of filtrates were combined and diluted to 250 mL with 75% ethanol and filtered through a 0.22 *μ*m microporous membrane. A 5 *μ*L aliquot of the resulting sample was injected into the HPLC-Q-TOF/MS for analysis.

### 2.3. Preparation of Blood Sample

Eight male Wistar rats of approximately (200 ± 20) g were obtained from the Laboratory Animal Center of Tianjin University of Traditional Chinese Medicine (Tianjin, China) and randomly separated into a control group (4 rats) and an SMW dosage group (4 rats). The animals were acclimatized to the facilities for 7 d and subjected to fasting with free access to water for a 12 h/12 h dark/light cycle prior to the experiment. SMW was orally administered to the rats at a dosage of 4.32 g/kg body weight. Two hours after drug administration, blood was collected from the ophthalmic veins of the rats by a sterile capillary tube and centrifuged at 6,000 ×g for 15 min. The supernatant (i.e., the serum) obtained was frozen immediately and stored at −20°C and thawed before analysis.

For GC-MS, 1 mL of acetonitrile was added into 400 *μ*L of the serum sample using a repeater pipette, vortexed for 60 s, and then centrifuged at 10,000 ×g for 10 min at 4°C. The supernatant was pipetted into a fresh tube and evaporated to dryness using nitrogen gas. The dried residue was dissolved in 100 *μ*L of acetonitrile before GC-MS analysis. For LC-MS, 1 mL of methanol and 100 *μ*L of potassium dihydrogen phosphate (1 mol/L) were added to 200 *μ*L of the serum sample to increase the extraction rate of alkaloids. The dried residue was dissolved in 100 *μ*L reconstituted solvent (methanol : water; 1 : 1, v/v) prior to LC-MS analysis.

### 2.4. Instrumentation and Conditions

#### 2.4.1. GC-MS Analysis

A Shimadzu QP2010 GC-MS system was used for the analysis using a DB-17MS fused-silica capillary column (30 m × 0.25 mm i.d., 0.25 *μ*m, Agilent, USA). The injector temperature was 250°C. The temperature programme used was as follows: initial maintenance at 100°C for 2 min, increase to 140°C at 10°C/min, held for 2 min, increase to 170°C at 2.5°C/min, held for 6 min, and then increase to 250°C at 8°C/min, held constant for 5 min. The electron energy and electron current were set to 70 eV. The ion source and interface temperatures were 200°C and 280°C, respectively. Chromatograms were first recorded in full-scan mode (33 amu to 550 amu) to identify the analytes and their respective retention times.

#### 2.4.2. LC-MS Analysis

Two different instruments and conditions were selected to meet different requirements. Q-TOF-MS has a high resolution and can yield the accurate molecular weights of compounds with which to infer their molecular formula, making it very useful for identifying unknown compounds. Thus, Q-TOF-MS was selected to analyze the chemical components of SMW in vitro. In vivo, however, the metabolites of the chemical components could be coupled with other functional groups. Thus, while MS^2^ spectra are useful, MS^3^ spectra are also necessary. Since Q-TOF-MS can only provide MS^2^ spectra, LCQ-MS, which can provide MS^3^ spectra, was employed.

In vitro LC-MS measurements were performed using an ESI-Q-TOF mass spectrometer (Bruker Daltonics, Germany) equipped with an Agilent HPLC system (Agilent, USA). Data Analysis 2.0 (Bruker Daltonics, Germany) was used to process the mass spectra obtained. Separation was performed on an Agilent SB-Aq column (2.1 mm × 100 mm, i.d., 1.8 *μ*m, Agilent, USA) with a column temperature set at 30°C. Mobile phase A was composed of water modified with 0.1% formic acid, while mobile phase B was composed of acetonitrile modified with 0.1% formic acid. The linear gradient was as follows: 0 to 6 min, B at 10% to 25%; 6 to 20 min, B at 25% to 38%; 20 to 25 min, B at 38% to 55%; 25 to 35 min, B at 55% to 80%; 35 to 40 min, B at 80% to 90%; 40 to 45 min, B at 90%, 45 to 47, B at 90% to 10%; 47 to 65 min, B at 10%. The flow rate was 0.2 mL/min and the injection volume was 5 *μ*L. Mass spectra were acquired in both the positive and negative modes. The capillary voltage was set to 4500 V in the positive mode and −2600 V in the negative mode, with an end plate offset potential of −500 V. The scan range was from 100* m/z* to 1000* m/z* with an acquisition rate of 1 spectrum per second both in the positive and negative mode. Dry gas was set to 6 L/min at 180°C with a nebulization gas pressure of 0.8 bar.

In vivo LC-MS analysis was carried on a Thermo Finnigan Surveyor LC and LCQ Deca XP Max mass spectrometer (Thermo Finnigan, San Jose, CA, USA) equipped with Finnigan Xcalibur 1.3 controlling software. An Agela Venusil XBP C_18_ column (2.1 mm × 150 mm, i.d., 5 *μ*m, Agela, China) was used to achieve separation with a gradient elution between mobile phase A (Formic acid : water = 0.1 : 100) and B (Formic acid : Acetonitrile = 0.1 : 100) at a flow rate of 0.2 mL/min. The column temperature was set at 30°C and the injection volume was 5 *μ*L. The elution gradients are as follows: start at 10% B, 20% B (16 min), 35% B (30 min), 90% B (45 min), and then return to 10% B (47 min). A reequilibration time of 18 min was used. Mass spectra were acquired in the positive mode with an ion spray voltage of 4.5 kV, capillary temperature of 300°C, sheath gas of 35 arb, and auxiliary gas of 5 arb. The mass range was set at 150* m/z* to 800* m/z* for higher sensitivity.

## 3. Results and Discussion

### 3.1. Analysis of Chemical Constituents

#### 3.1.1. Identification of Chemical Components in Volatile Extracts

**Figure 1:**
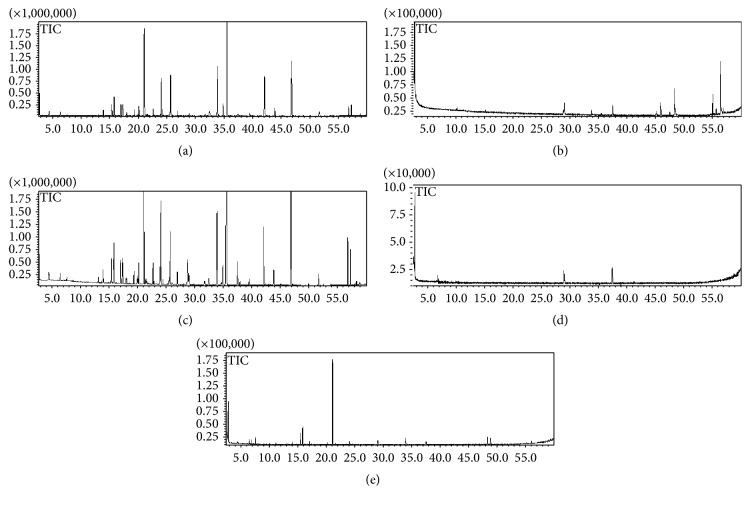
Total ion chromatograms obtained by GC-MS of volatile oils from (a) SMW and its major constituent herbs, (b)* Cortex Phellodendri*, (c)* Rhizoma Atractylodis*, (d)* Radix Achyranthis Bidentatae*, and (e)* Semen Coicis*.

The total ion chromatograms (TIC) of volatile extracts from SMW and its four constituent herbs are shown in Figure 1. All the main components were completely separated at 65 min. Figure 1 shows that most of the volatile extracts of SMW are from* Rhizoma Atractylodis*. Of all the compounds detected by GC-MS, 26 compounds were identified by searching the NIST2008 and WILEY databases (through Chemstation) and comparing their retention times and MS spectra with the reference literature [6–11].  Table 1 shows the identification result and the percentages of identified compounds.

#### 3.1.2. Identification of Chemical Components in Nonvolatile Extracts

**Figure 2:**
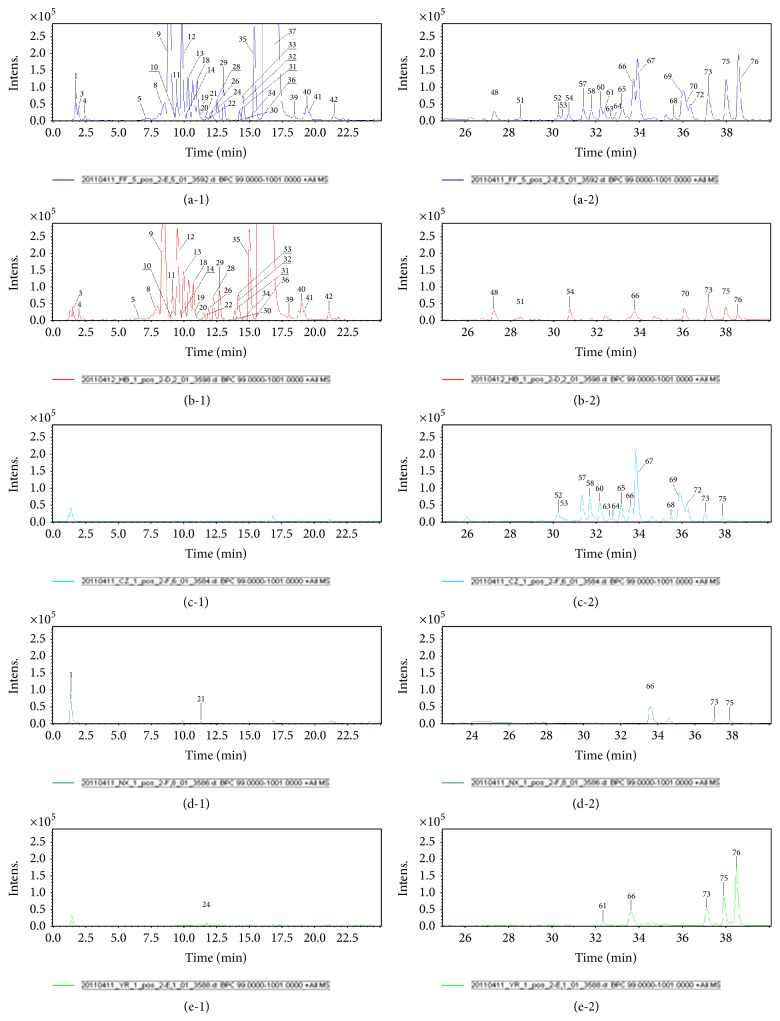
Base peak chromatograms obtained by RPLC/Q-TOF-MS in the positive mode of nonvolatile extracts from SMW and its constituent herbs. (Chromatograms at 0 to 25 min: (a-1) SMW; (b-1)* Cortex Phellodendri*; (c-1)* Rhizoma Atractylodis*; (d-1)* Radix Achyranthis Bidentatae*; (e-1)* Semen Coicis*; Chromatogram at 25–40 min: (a-2) SMW; (b-2)* Cortex Phellodendri*; (c-2)* Rhizoma Atractylodis*; (d-2)* Radix Achyranthis Bidentatae*; (e-2)* Semen Coicis*).

**Figure 3:**
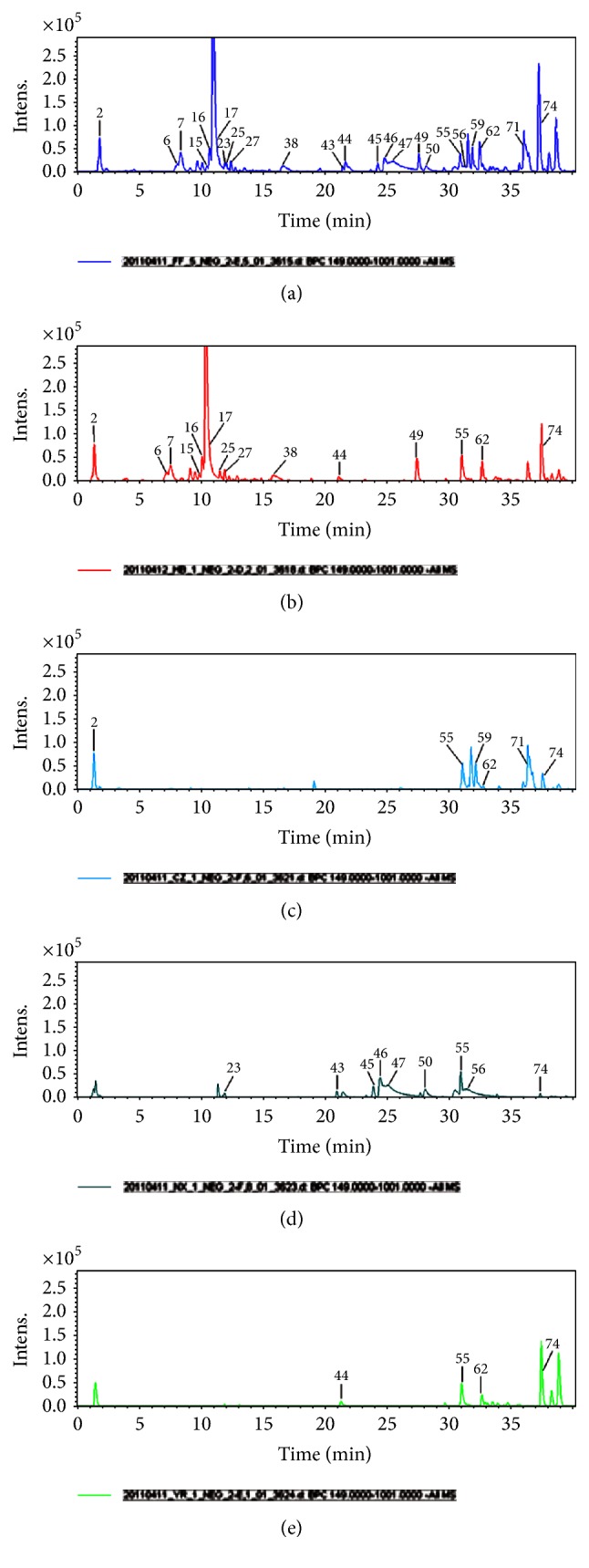
Base peak chromatograms obtained by RPLC/Q-TOF-MS in the negative mode of nonvolatile extracts from SMW and its constituent herbs. (a) SMW; (b)* Cortex Phellodendri*; (c)* Rhizoma Atractylodis*; (d)* Radix Achyranthis Bidentatae*; (e)* Semen Coicis*.

**Figure 4:**
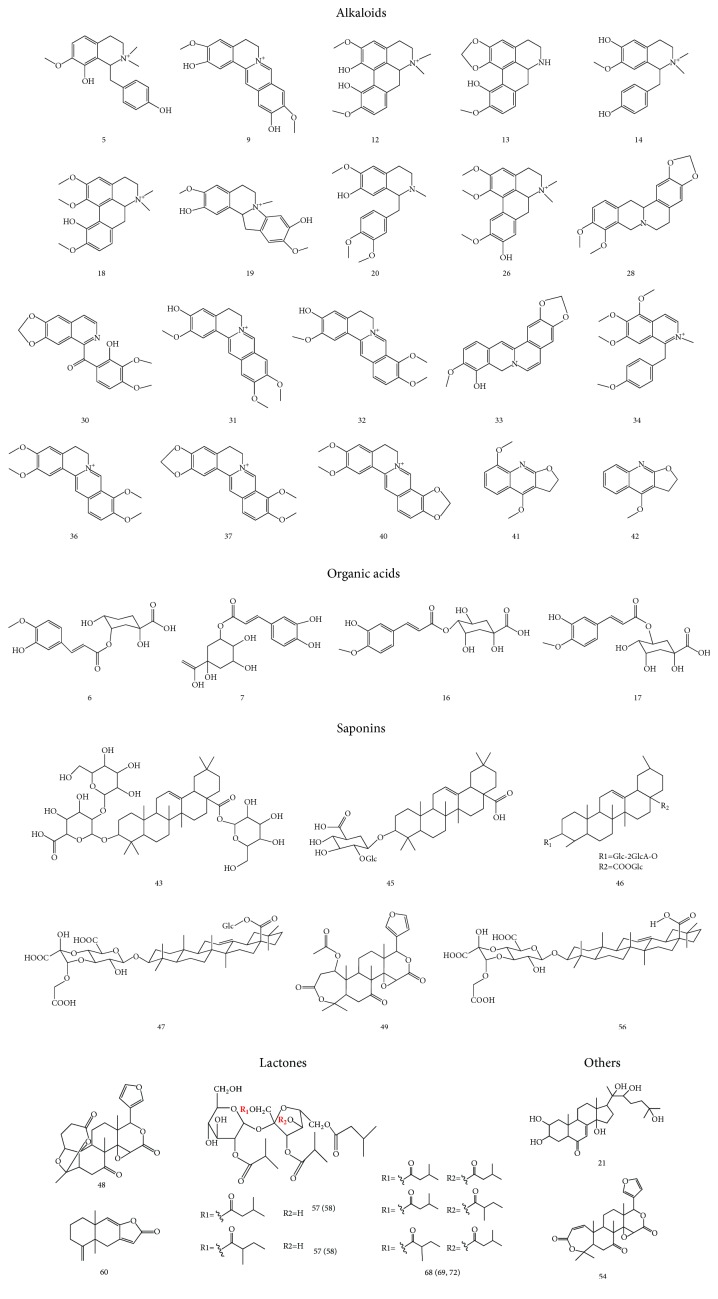
Structures of compounds identified in the nonvolatile extracts from SMW and its constituent herbs.

Mass spectra were acquired both in the positive and negative ion modes. Figures 2 and 3 show base peak chromatograms (BPC). A total of 76 main compounds were elucidated from the nonvolatile extracts, 49 of which were identified. Structures of the main compounds are shown in Figure 4 and the fragment ions and identification results are listed in Table 2. Comparing the BPC of SMW with those of the constituent herbs, 42 compounds were found and assigned to* Cortex Phellodendri* (peaks 2–20, 22, 25–42, 48-49, 51, 54, and 70),* Rhizoma Atractylodis* (peaks 52-53, 57–60, 63–65, 67–69, and 71-72),* Radix Acanthopanacis Bidentatae* (peaks 1, 23, 43, 45–47, 50, and 56), and* Semen Coicis* (peaks 24 and 61). Peaks such as those at 44 and 76 were obtained from both* Cortex Phellodendri* and* Semen Coicis*, while peaks 55, 66, 73, and 75 were found in all the constituent herbs.

In the positive ion mode, [M]^+^, [M + H]^+^, [M + Na]^+^, and [2M + Na]^+^ were observed, while [M − H]^−^ and [M + HCOO]^−^ were commonly seen in the negative ion mode. The information from [M]^+^, [M + H]^+^, [M + Na]^+^, [2M + Na]^+^, [M − H]^−^, and [M + HCOO]^−^ was used to determine molecular weights, while MS/MS data were used to determine the structures of the compounds involved. The compounds detected were divided into several categories, like alkaloids, organic acids, saponins, lactones, and so on.

*Identification of Alkaloids*. All the alkaloids detected were obtained from* Cortex Phellodendri*, consistent with literature reports. Peaks 5 and 14 had the same [M]^+^ ion at* m/z* 314, but exhibited different fragment ions in MS/MS spectra. The fragment ions (*m/z* 283 [M − CH_3_  − H − CH_3_]^+^ and* m/z* 298 [M − CH_3_  − H]^+^) of peak 5 were observed, while peak 14 showed* m/z* 164 [M − CH_3_  − C_7_H_7_O − CO]^+^,* m/z* 192 [M − CH_3_  − C_7_H_7_O]^+^,* m/z* 239 [M − H − CO − 2CH_3_]^+^, and* m/z* 269 [M − H − CO]^+^ in the MS/MS spectra. Comparison with the literature data [12] identified peaks 5 and 14 as (−)-oblongine and lotusine. Peaks 9 and 12 were isomers because they yielded the same [M]^+^ ion at* m/z* 342. They were identified as phellodendrine and magnoflorine, respectively, by comparison with literature data [12–14] and reference standard compounds. The main MS/MS fragment ion of peak 9 at* m/z* 342 was* m/z* 192, which is generated because of RDA rearrangement. Peaks 18 and 26 had the same molecular formula and similar MS/MS spectra, indicating that their structures are similar. Literature data [12] showed peaks 18 and 26 as menispermine and xanthoplanine, respectively, which differ only in the position of the hydroxyl group. For peaks 29, 31, 32, 36, 37, 40, and 51, the fragment ions* m/z* [M − CH_3_  − H]^+^ and* m/z* [M − CH_3_  − H − CH_2_  − CO]^+^ were observed in their MS/MS spectra; these compounds were identified as protoberberine alkaloids. Some peaks had the same [M]^+^ ion. For example, peaks 29, 36, and 51, peaks 31 and 32, peaks 33 and 35, and peaks 37 and 40 had the same* m/z* values in the MS spectra and similar fragment ions in the MS/MS spectra. Comparison with the reference standard compounds and literature data [12–15] identified peak 36 as palmatine and peaks 29 and 51 as isomers of palmatine. Similarly, peaks 31, 32, 33, 35, 37, and 40 were identified as columbamine, jateorhizine, berberubine, tetradehydroscoulerine (or tetrahydrocheilanthifolinium), berberine, and epiberberine [12–15], respectively. For peaks 19, 28, 41, and 42, the base peak [M + H]^+^ ion was observed in the MS spectra. Comparison of the retention times, MS spectra, and MS/MS spectral data with those of reference compounds reported in literature [12, 13] associated the peaks with litcubine, tetrahydrocoptisine, *γ*-fagarine, and dictamnine, respectively. The corresponding data of peaks 13, 20, 30, and 34 were not found in the literature of* Cortex Phellodendri*; by analyzing their MS and MS/MS data, they were temporarily considered as cassythidine, codamine, rugosinone, and takatonine. The study of these compounds is ongoing.

*Identification of Organic Acids*. Organic acids in the present study had better responses in the negative ion mode. The full mass spectra of peaks 6, 16, and 17 showed similar deprotonated molecule ions [M − H]^−^ at* m/z* 367, but they could be set apart by their MS/MS spectra. The MS/MS spectra of peak 6 indicated a characteristic ion at* m/z* 193 [ferulic acid − H]^−^, while peaks 16 and 17 showed predominant ions at* m/z* 173 [quinic acid − H_2_O − H]^−^ and* m/z* 191 [quinic acid − H]^−^, respectively. Comparison with literature data [12, 13] identified peaks 6, 16, and 17 as 5-O-feruloylquinic acid, 4-O-feruloylquinic acid, and 3-O-feruloylquinic acid, respectively. Peak 7 exhibited a [M − H]^+^ ion at* m/z* 353 and its MS/MS spectra was observed at* m/z* 173 [M − H − caffeoyl − H_2_O]^−^ and* m/z* 191 [M − H − caffeoyl]^−^. Comparison with the literature data [12] identified peak 7 as caffeotannic acid or one of its isomers. Peak 15 had the same fragment ion at* m/z* 191 [quinic acid − H]^−^ as peak 17, hence it was considered as a derivative of quinic acid. 

*Identification of Saponins*. Peak 46 exhibited a molecular ion at* m/z* 955 [M − H]^−^, and fragmentation ions at* m/z* 793 [M − H − Glc]^−^,* m/z* 631 [M − H − 2Glc]^−^,* m/z* 569 [M − H − 2Glc − H_2_O − CO_2_]^−^, and* m/z* 455 [M − H − 2Glc − GlcA]^−^ were observed in its MS/MS spectra. Comparison with the literature data [16] identified peak 46 as ginsenoside Ro. Peaks 43 and 50 had identical molecular ions at* m/z* 955 [M − H]^−^, but different MS/MS fragmentation spectra and retention times in the chromatogram. Comparison of the retention times, MS and MS/MS data with literature data identified peaks 43 and 50 as chikusetsusaponin V [17] and isomer of ginsenoside Ro. In the same way, peaks 45, 47, 49, 56, and 67 were identified as zingibrosideR1 [18], achyranthis saponin III [19], nomilin [12], achyranthis saponin IV [19], and (2E, 8E)-2,8-decadiene-4,6-diyne-1,10-diol-1-*β*-D-glycopyranoside [20, 21], respectively. 

*Identification of Lactones*. Comparison of the retention times and MS and MS/MS data with the reference standard compounds identified peak 48 as obakulactone [22]. Peaks 57 and 58 were isomers that showed the same quasimolecular ion at* m/z* 701 [M + Na]^+^; they were both identified as atractysucrose III [23]. Peaks 68, 69, and 72 were also isomers labeled as atractysucrose I [23]. Comparison of the MS and MS/MS data with the reported literature data [24] identified peak 60 as atractylenolide I. 

*Identification of Other Compounds*. In the MS spectra, peak 54 showed molecular ions [M + H]^+^ at* m/z* 455 and was identified as obakunone, after comparison with the literature data. Peak 21 was identified as ecdysterone [25] by direct comparison with the standard compound.

### 3.2. Analysis of the Components Absorbed into Blood

#### 3.2.1. Identification of Components in Rat Plasma Based on GC-MS

**Figure 5:**
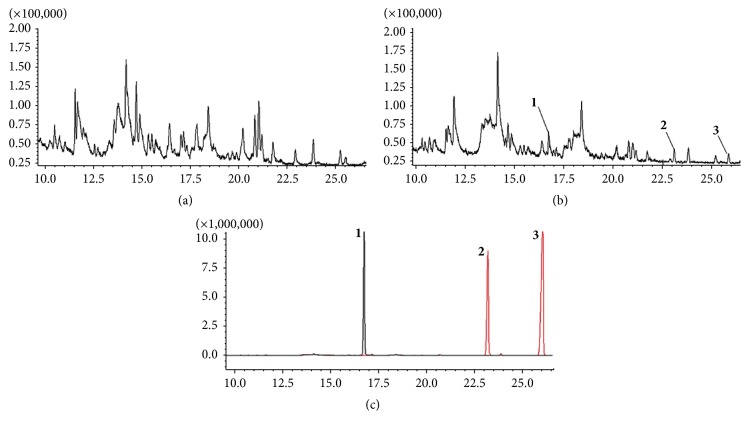
Total ion chromatograms obtained by GC-MS of (a) control serum, (b) serum after oral administration of SMW, and (c) reference substance.

As shown in Figure 5, three peaks were observed in dosed rat plasma but not in the controlled rat plasma. Of the three peaks, 1 and 3 were found to be absorbed in the prototype form; these compounds were identified as *β*-eudesmol and atractylodine by comparison with the literature data and reference standards. The MS and MS/MS fragmentation data of peak 2 were identical to the decomposition products of atractylodine. Therefore, peak 2 may be a metabolite of atractylodine in vivo.

#### 3.2.2. Identification of Components in Rat Plasma Based on LC-MS

**Figure 6:**
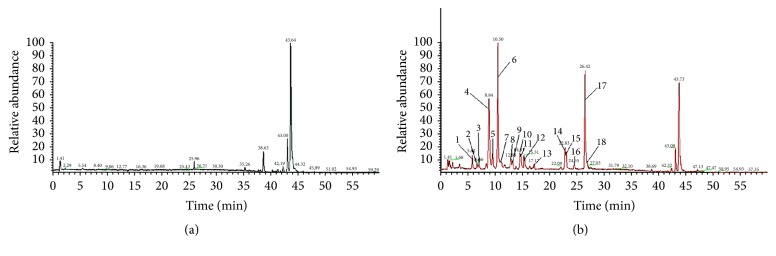
Extracted ion chromatogram obtained by HPLC-MS of (a) control serum and (b) serum after oral administration of SMW.

The chromatograms were collected under optimized conditions.  Figure 6 shows typical extracted ion chromatograms of the control serum sample and drug-containing serum sample in positive ion mode. A total of eighteen peaks were observed in the drug-containing serum but not in the control serum. As a result, peaks 4, 6, 9, 12, 14, 16, and 18 were found to be absorbed by blood in the prototype form. Peaks 10, 11, 15, and 16 were tentatively identified as metabolites. Peaks 1, 2, 3, 5, 7, 8, and 13 could not be positively identified in the present study. Detailed information of the constituents absorbed in rat plasma is shown in Table 3.

Both peaks 4 and 6 have molecular ions [M + H]^+^ at* m/z* 342, but their MS/MS fragment data are different. Comparison with the reference standards and literature data identified peaks 4 and 6 as phellodendrine and magnoflorine [12], respectively. Peak 9 showed a molecular ion at* m/z* 314 [M + H]^+^ and was considered to be either oblongine or lotusine [12]. Peak 12 exhibited molecular ions [M]^+^ at* m/z* 356 and MS/MS fragments ions at* m/z* 311 and 279. According to the literature data, peak 12 was considered to be dauricine. Peaks 14, 17, and 18 displayed molecular ions [M + H]^+^ (peak 14), [M]^+^ at* m/z* 322, 336, and 352, respectively. Similar fragment ions* m/z* [M − CH_3_]^+^ and* m/z* [M − CH_3_  − CO]^+^ were observed in their MS/MS spectra. Based on the above observations and comparisons with the reference standards and literature date, peaks 14, 17, and 18 were identified as berberubine, berberine, and palmatine [13], respectively.

Peaks 10, 11, 15, and 16 were identified as metabolites of prototype compounds. Peak 10 exhibited a molecular ion [M]^+^ at* m/z* 514. In the MS^2^ spectra of the [M]^+^ ion, the peak due to the loss of a glucuronyl at* m/z* 338 was detected (−176 Da C_6_H_9_O_7_), and the MS^3^ spectra of ion 338 were identical to the MS^2^ spectra of reference standard jateorhizin. Therefore, peak 10 was identified as jateorhizine-3-O-*β*-D-glucuronide. Similarly, peaks 11, 15, and 16 were temporarily identified as berberubine-9-O-*β*-D-glucuronide, a metabolite of berberine, and berberubine-9-O-sulfate. Peaks 1, 2, 3, 5, 7, 8, and 13 could not be identified in the present study. Work on these peaks is ongoing.

## 4. Conclusions

A comprehensive and systematic analytical method was established for the first time to analyze the components of SMW. In the current study, numerous constituents in SMW and rat plasma after oral administration of SMW were successfully identified by the combined GC-MS and LC-MS method. Volatile oils, alkaloids, organic acids, saponins, and lactones were all found in the SMW extracts. Of all the chemicals identified, alkaloids from* Cortex Phellodendri* could be the main bioactive compounds in SMW. The current research provides a reliable supplement for the quality control and pharmacological study of SMW. The proposed method was not only appropriate for the rapid screening and identification of multiple components in the extract of SMW but also a good method for screening its bioactive constituents in vivo. The method presented in the present work could be a potentially useful tool in the study of other herbs.

## Figures and Tables

**Table 1:** 26 compounds identified in volatile oils from SMW and its constituent herbs.

Number	RT (min)	Compounds	Molecular formula	Molecular weight	%
1	2.78	Methylbenzene	C_7_H_8_	92	1.43
2	4.43	*α*-Pinene	C_10_H_16_	136	0.68
3	6.45	1-Phellandrene	C_10_H_16_	136	0.49
4	7.58	1-Methyl-2-(1-Methylethyl)-benzene	C_10_H_14_	134	0.1
5	13.07	1,3,4,5,6,7-Hexahydro-2,5,5-trimethyl-2H-2,4a-ethanonaphtalene	C_15_H_24_	204	0.16
6	15.41	Longifden	C_15_H_24_	204	1.22
7	15.83	4,4-Dimethyl-adamantan-2-ol	C_12_H_20_O	180	2.2
8	16.99	*δ*-Guaiene	C_15_H_24_	204	1.5
9	17.34	Trans-caryophellene	C_15_H_24_	204	1.77
10	18.02	*α*-Caryophellene	C_15_H_24_	204	0.46
11	19.33	*α*-Cedrene	C_15_H_24_	204	0.75
12	20.17	*γ*-selinene	C_15_H_24_	204	1.18
13	21.10	*β*-selinene	C_15_H_24_	204	10.89
14	22.67	*β*-Sesquiphellandrene	C_15_H_24_	204	0.79
15	24.07	*γ*-selinene	C_15_H_24_	204	4.85
16	25.69	*γ*-Elemene	C_15_H_24_	204	5.75
17	26.90	*β*-Vatirenene	C_15_H_22_	202	0.58
18	32.48	Hinesol	C_15_H_26_O	222	0.83
19	33.91	*β*-Eudesmol	C_15_H_26_O	222	8.63
20	34.88	*α*-Bisabolol	C_15_H_26_O	222	2.03
21	35.53	Furanodiene	C_15_H_20_O	216	33.4
22	37.47	Diethyl phthalate	C_12_H_14_O_4_	222	0.35
23	46.83	Atrctylodin	C_13_H_10_O	182	8.58
24	56.82	Trans-tricyclo[8.6.0.0(2,9)]- 3,15-hexadecadiene	C_16_H_24_	216	0.54
25	57.27	5-Methyl-1-[2,6,6-Trimethyl-2,4-cyclohexadienyl]-1,4-hexadiene-3-one	C_16_H_22_O	230	0.73
26	58.85	Isolantolactonoid butenolide A	C_15_H_20_O_2_	232	0.13

**Table 2:** Characterization of compounds in SMW by RPLC-Q-TOF/MS.

Number	RT (min)	Ion mode	Characteristic ion	Fragment ions (*m*/*z*)	Identification	Origin
1	1.77	+	118.0851	—	Valine	C
2	1.86	−	191.0650	—	Quinic acid	B, C
3	1.94	+	192.1011	133.05, 148.07, 176.05	—	A
4	2.47	+	180.1333	—	—	A
5	7.31	+	314.1678	283.08 [M − CH_4_ − CH_3_]^+^, 298.10 [M − CH_4_]^+^	(−)-oblongine	A
6	8.12	−	367.1033	193.06 [ferulic acid − H]^−^	5-O-feruloylquinic acid	A
7	8.38	−	353.0905	179.04 [caffeic acid − H]^−^, 191.07 [quinic acid − H]^−^	Caffeotannic acid	A
8	8.39	+	448.1959	143.05, 178.09, 255.10, 286.14	—	A
9	8.88	+	342.1741	192.10 [M − C_9_H_10_O_2_]^+^, 177.07 [M − C_9_H_10_O_2_ − CH_3_]^+^	Phellodendrine	A
10	9.35	+	328.1827	166.09, 252.09, 312.12	—	A
11	9.44	+	592.2408	178.08, 255.10, 286.14	—	A
12	9.82	+	342.1685	265.08 [M − C_2_H_7_N − CH_3_ − CH_3_OH]^+^, 282.02 [M − C_2_H_7_N − CH_3_]^+^, 297.11 [M − C_2_H_7_N]^+^	Magnoflorine	A
13	9.99	+	312.1223	177.08 [M + H − C_7_H_4_O_2_ − CH_3_]^+^, 240.10 [M + H − CH_3_ − CHO − CO]^+^, 268.09 [M + H − CH_3_ − CHO]^+^	Cassythidine	A
14	10.29	+	314.177	192.10 [M − CH_3_ − H − C_7_H_7_O + H]^+^, 239.07 [M − H − CO − 2CH_3_]^+^, 269.11 [M − H − CO]^+^	Lotusine	A
15	10.47	−	337.0939	191.06 [quinic acid − H]^−^, 163.04	Derivation of Quinic acid	A
16	10.69	−	367.1084	173.05 [quinic acid − H_2_O − H]^−^	4-O-feruloylquinic acid	A
17	10.97	−	367.1075	191.07 [quinic acid − H]^−^	3-O-feruloylquinic acid	A
18	10.991	+	356.1873	192.10, 177.08, 265.08	Menispermine	A
19	11.10	+	328.1884	121.06, 208.08	Litcubine	A
20	11.12	+	344.1842	137.06 [M − C_11_H_14_NO_2_ − CH_3_ + H]^+^, 192.09 [M − C_9_H_11_O_2_ + H]^+^	Codamine	A
21	11.58	+	481.3129	173.10, 371.22 [M + H − 2H_2_O − C_4_H_10_O]^+^	Ecdysterone	C
22	11.78	+	350.0995	279.05, 294.07, 322.07	—	A
23	11.89	−	525.3078	159.11, 319.20	—	C
24	11.95	+	438.2375	119.05, 147.04, 204.10, 275.17		D
25	11.99	−	567.2102	314.13, 329.15	—	A
26	12.14	+	356.185	177.08, 192.10	Xanthoplanine	A
27	12.37	−	679.2255	219.08, 337.13	—	A
28	12.53	+	324.1229	266.08 [M − CH_2_ − 2H + H − CH_2_]^+^, 280.09 [M − CH_2_ − 2H − CO + H]^+^, 308.09 [M − CH_2_ − 2H + H]^+^	Tetrahydrocoptisine	A
29	13.01	+	352.119	294.07 [M − CH_3_ − H − CH_2_ − CO]^+^, 308.09 [M − CH_3_ − H − CO]^+^, 336.08 [M − CH_3_ − H]^+^	Isomer of palmatine	A
30	14.19	+	354.0953	320.06 [M + H − H_2_O − CH_3_ − H]^+^, 336.04 [M + H − H_2_O]^+^	Rugosinone	A
31	14.22	+	338.1376	280.09 [M − CH_3_ − H − CH_2_ − CO]^+^, 307.08 [M − CH_3_ − H − CH3]^+^, 322.11 [M − CH_3_ − H]^+^	Columbamine	A
32	14.50	+	338.1387,	280.09 [M − CH_3_ − H − CH_2_ − CO]^+^, 307.08 [M − CH_3_ − H − CH_3_]^+^, 322.10 [M − CH_3_ − H]^+^	Jateorhizine	A
33	14.51	+	322.1067	279.09 [M + H − CH_3_ − CO]^+^, 307.08 [M + H − CH_3_]^+^	Berberubine	A
34	14.90	+	354.1677	190.08	Takatonine	A
35	15.34	+	322.1104	279.09 [M − CH_3_ − CO]^+^, 307.08 [M − CH_3_]^+^	Tetradehydroscoulerine	A
36	15.89	+	352.117	294.07 [M − CH_3_ − H − CH_2_ − CO]^+^, 308.12 [M − CH_3_ − H − CO]^+^, 322.07 [M − CH_3_ − H − CH_2_]^+^, 336.11 [M − CH_3_ − H]^+^,	Palmatine	A
37	16.34	+	336.1261	278.08 [M − CH_3_ − H − CH_2_ − CO]^+^, 292.10 [M − CH_3_ − H − CO]^+^, 306.08 [M − CH_3_ − H − CH_2_]^+^, 320.09 [M − CH_3_ − H]^+^	Berberine	A
38	16.63	−	426.1202	—	—	A
39	18.39	+	350.1370	292.09, 306.10, 320.09, 334.10		A
40	19.34	+	336.1217	320.09 [M − CH_3_ − H]^+^, 278.08 [M − CH_3_ − H − CH_2_ − CO]^+^	Epiberberine	A
41	19.53	+	230.08	144.04, 172.04, 200.03	*γ*-Fagarine	A
42	21.39	+	200.0697	129.05, 185.04	Dictamnine	A
43	21.58	−	955.4855	793.48 [M − H − Glc]^−^, 569.41 [M − H − 2Glc − H_2_O − CO_2_]^−^, 523.40 [M − H − 2Glc − 2H_2_O − CO_2_]^−^	Chikusetsusaponin V	C
44	21.77	−	329.2349	211.14	—	A, D
45	24.43	−	793.43.65	455.37 [M − H − Glc − GlcA]^−^, 570.41 [M − 2Glc − H_2_O − CO_2_]^−^, 631.41 [M − H − Glc]^−^	Zingibroside R1	C
46	25.13	−	955.4419	455.37 [M − H − 2Glc − GlcA]^−^, 631.42 [M − H − 2Glc]^−^, 673.43, 793.48 [M − Glc]^−^, 835.49	Ginsenoside Ro	C
47	25.48	−	953.9747	455.37, 569.41, 631.41, 793.48, 835.48	3-O-(3′-carboxymethoxyl-3-oxyacetone acid-3′-acetal-4′-hemiketal)-*β*-glucuronyl-oleanolic acid-28-O-*β*-D-glucopyranoside	C
48	27.36	+	471.1995	161.06, 213.09, 425.19	Obakulactone	A
49	27.66	−	515.196	229.13	Nomilin	A
50	28.27	−	955.4417	455.37 [M − H − 2Glc − GlcA]^−^, 613.40 [M − H − 2Glc − H_2_O]^−^, 793.48 [M − H − Glc]^−^, 835.49	Isomer of ginsenoside Ro	C
51	28.53	+	352.1152	294.07 [M − CH_3_ − H − CH_2_ − CO]^+^, 322.07 [M − CH_3_ − H − CH_2_]^+^	Isomer of Palmatine	A
52	30.30	+	255.1344	—		B
53	30.46	+	283.1302	—		B
54	30.75	+	455.2074	161.07, 183.07	Obakunone	A
55	31.00	−	313.2416	185.13	—	A, B, C, D
56	31.34	−	791.3842	455.38, 631.41	Achyranthis saponin IV	C
57	31.42	+	701.3396	251.08, 455.22	Atractysucrose-III	B
58	31.93	+	701.3385	—	Atractysucrose-III	B
59	32.00	−	677.3414	—	—	B
60	32.24	+	231.1385	128.06, 141.06, 155.08	Atractylenolide I	B
61	32.39	+	319.2237	—	—	D
62	32.60	−	295.2305	—	—	A, B, D
63	32.68	+	313.1398	—	—	B
64	32.85	+	381.1657	128.06, 52.06, 178.07	—	B
65	33.22	+	309.1098	141.07, 165.07	—	B
66	33.73	+	301.1425	—	—	A, B, C, D
67	33.91	+	325.1433	121.03, 149.02, 167.08	(2E, 8E)-2,8-decadiene-4,6-diyne-1,10-diol-1-*β*-D-Glycopyranoside	B
68	35.57	+	785.3907	251.08, 455.22	Atractysucrose-I	B
69	36.03	+	785.3965	233.07, 335.14, 437.21, 539.27	Atractysucrose-I	B
70	36.05	+	279.2315	—	—	A
71	36.14	−	807.4036	—	—	B
72	36.32	+	785.3951	—	Atractysucrose-I	B
73	37.15	+	303.2298	—	—	A, B, C, D
74	37.35	−	279.2378	—	—	A, B, C, D
75	37.96	+	257.2491	—	Hexadecanoic acid	A, B, C, D
76	38.53	+	283.2655	—	Oleic acid or Petroselinic acid or Octadecylic acid	A, D

A: *Cortex Phellodendri*; B: *Rhizoma Atractylodis*; C: *Radix Acanthopanacis Bidentatae*; D: *Semen Coicis*.

**Table 3:** MS/MS data of (+) ESI-MS spectra and the identification results of the constituents of SMW.

No.	RT (min)	[M]^+^ or [M + H]^+^ (*m*/*z*)	MS^2^ (*m*/*z*)	MS^3^ (*m*/*z*)	Identified compounds
1	5.82	450.5	234.8, 217.0		Not identified
2	6.66	404.0	193.0, 210.9		Not identified
3	6.96	328.0	309.9, 265.2, 283.0		Not identified
4	8.84	342.0	192.2		Phellodendrine
5	9.49	448.0	286.1		Not identified
6	10.49	342.0	297.1, 265.2		Magnoflorine
7	11.09	558.0	525.0, 472.0		Not identified
8	12.90	369.0	177.1		Not identified
9	13.23	314.5	269.0		Oblongine or lotusine
10	14.51	514.0	338.2	265.1, 307.1, 322.1	Jateorhizine-3-O-*β*-D-glucuronide
11	14.71	498.0	322.1		Berberubine-9-O-*β*-D-glucuronide
12	15.34	356.0	311.0, 279.1		Dauricine
13	17.19	540.0	331.0, 471.1		Not identified
14	22.81	322.0	307.3, 322.2		Berberubine
15	23.04	354.0	336.2	321.2 292.4	Metabolite of berberine
16	24.53	402.0	322.1	307.3	Berberubine-9-O-sulfate
17	26.42	336.0	321.2, 292.4		Berberine
18	26.92	352.0	337.2, 308.3		Palmatine
